# COVIDomics: Metabolomic Views on COVID-19

**DOI:** 10.3390/metabo14120702

**Published:** 2024-12-12

**Authors:** Armando Cevenini, Lucia Santorelli, Michele Costanzo

**Affiliations:** 1 Department of Molecular Medicine and Medical Biotechnology, University of Naples Federico II, 80131 Naples, Italy; armando.cevenini@unina.it; 2 CEINGE–Biotecnologie Avanzate Franco Salvatore, 80145 Naples, Italy; 3 Department of Oncology and Hematology-Oncology, University of Milan, 20122 Milan, Italy; luciasantorelli13@gmail.com

During the COVID-19 pandemic, omics-based methodologies were extensively used to study the pathological mechanisms of SARS-CoV-2 infection and replication in human cells at a large scale [1,2,3,4,5,6]. These approaches supported diverse goals, including diagnostics, disease monitoring, drug target discovery, and vaccine development and safety. In fact, omics enabled a deeper understanding of the biochemical and pathophysiological mechanisms underlying SARS-CoV-2 infection and transmission, providing strategies to control the virus and manage disease outcomes [1,2,4,6,7,8].

As omics datasets related to COVID-19 research have exponentially increased over the last four years, we employed the term COVIDomics to include all the scientific efforts from the community. Among the omic technologies, metabolomics provides insights into the biochemical landscape within pathological contexts [9,10,11,12,13,14,15,16]. Metabolomics and lipidomics approaches in biomedical research are commonly based on the latest high-resolution liquid chromatography-tandem mass spectrometry (LC-MS/MS) platforms; however, certain analyses are performed using gas chromatography (GC)-MS or nuclear magnetic resonance (NMR) [16,17,18,19,20,21].

The findings revealed by metabolomics include the identification of key metabolic pathways, such as amino acid and lipid metabolism, as well as aspects of energy production and immune-related processes, which undergo significant changes in COVID-19 patients, particularly in those hospitalized with severe disease. This metabolic shift highlights potential therapeutic targets to modulate disease severity and immune response in COVID-19. Metabolic reprogramming has become recognized as a hallmark of COVID-19 infection, driven by SARS-CoV-2 to facilitate its replication and further altered by the immune response of the host organism [4,22,23,24,25,26,27,28].

The insights gained from these metabolomic findings underscore the complex interplay between viral infection and host metabolic reprogramming in COVID-19. As researchers seek ways to monitor and influence these biochemical changes, attention has turned to innovative diagnostic methods that allow for the dynamic tracking of disease markers over time. In this context, liquid biopsy emerges as a minimally invasive approach for accessing critical physiological information [29]. Bodily fluids (such as urine, blood, and plasma) present a promising way to uncover otherwise elusive molecular indicators of disease, including the systemic conditions induced by SARS-CoV-2 infection.

This Special Issue brings together six published articles that highlight the impact of SARS-CoV-2 on the human metabolism, with a particular focus on changes in biofluids (plasma, urine, saliva, and exhaled air), providing instrumental insights for diagnosing, monitoring, and treating COVID-19 and its long-term effects even in the post-acute sequelae of SARS-CoV-2 (PASC or long-COVID) [30]. Most of these authors highlighted the diagnostic potential of metabolomics and found that some metabolomic changes can be evaluated as potential biomarkers through the identification of unique metabolic profiles using MS or NMR spectroscopy. However, the methodological inconsistencies across studies underscore the need for the standardization and harmonization of large-scale metabolomics to enhance its value and applicability [31]. Bourgin et al. dealt with this particular topic in their review article, underlying that standardization is essential for integration into clinical settings [32].

Research articles on COVIDomics aim to individuate biomarkers of severity, the molecules that can inhibit SARS-CoV-2, or the metabolic alterations that could possibly be targeted for treatment. Rosolanka et al. analyzed the metabolomic changes in the urine of COVID-19 patients during the acute phase and in the recovery period after one month of infection. They found a urinary increase in acetone, carnitine, and citrate after hospital admission, connecting this with a switch of energy metabolism from glycolysis/Krebs cycle to ketosis, which was resolved after recovery. The reduction in the hippurate levels did not recover after one month, suggesting that the persistent suppression of the gut microbiota was induced by antibiotics. However, several other amino acids did not recover as well. Their results show that the metabolomic anomalies still endure one month after the infection, meaning that the organism is not fully recovered [33].

The complementary research of Liptak et al. showed that the blood metabolome alterations in acute COVID-19 patients also persist months after acute SARS-CoV-2 infection. The authors define a plasma metabolic signature that includes elevated glucose and 3-hydroxybutyrate levels and reduced acetate and histidine levels with the normalization of branched-chain amino acids and branched-chain keto acids [34].

Elkaeed et al. screened 4924 African natural products using multistage computational methods to identify potential SARS-CoV-2 inhibitors. By means of computational chemistry techniques, including molecular docking, toxicity, and molecular dynamics simulation experiments, the authors identified four metabolites—hippacine, 4,2′-dihydroxy-4′-methoxychalcone, 2′,5′-dihydroxy-4-methoxychalcone, and wighteone—that are able to bind the papain-like protease (PLpro) of SARS-CoV-2 with possible inhibitory effects [35].

Guntur et al. investigated exercise intolerance in PASC, which involves alterations in the metabolism and mitochondrial dysfunction. Plasma metabolomics revealed that PASC patients have increased free- and carnitine-conjugated unsaturated fatty acids and lower levels of key metabolic intermediates (pyruvate, lactate, citrate, succinate, and malate), polyamines (spermine) and taurine. Persistent tryptophan reduction, a feature of disease severity in COVID-19, is not recovered in PASC patients, suggesting ongoing metabolic disruption. These findings of at rest-patients align with the mitochondrial dysfunctions seen during exercise, highlighting potential therapeutic avenues to restore mitochondrial function when improving fatty acid catabolism in PASC patients [36].

Huang and colleagues underscored that individuals with obesity or type 2 diabetes (T2D) exhibit specific metabolomic profiles that may predispose them to severe COVID-19 outcomes. Using Mendelian Randomization as the statistical approach, the authors identified human serum metabolites causally associated with COVID-19 susceptibility and severity. These metabolites, such as gamma-glutamyltyrosine, mediate the path from T2D/obesity to severe COVID-19, possibly affecting COVID-19 progression. Generally, such markers may be considered as potential targets for risk assessment and treatment [37].
